# Monoaminergic regulation of Sonic hedgehog signaling cascade expression in the adult rat hippocampus

**DOI:** 10.1016/j.neulet.2009.02.034

**Published:** 2009-04-10

**Authors:** Rajeev Rajendran, Shanker Jha, Kimberly A. Fernandes, Sunayana B. Banerjee, Farhan Mohammad, Brian G. Dias, Vidita A. Vaidya

**Affiliations:** Department of Biological Sciences, Tata Institute of Fundamental Research (TIFR), Homi Bhabha Road, Mumbai 400005, India

**Keywords:** Serotonin, Norepinephrine, p-Chlorophenylalanine, p-Chloroamphetamine, 5,7-Dihydroxytryptamine, DSP-4, Fluoxetine, Desipramine, Tranylcypromine

## Abstract

Monoamines are implicated in the modulation of adult hippocampal neurogenesis in depression models and following chronic antidepressant treatment. Given the key role of Sonic hedgehog (Shh) in adult neurogenesis, we examined whether monoaminergic perturbations regulate the expression of Shh or its co-receptors Smoothened (Smo) and Patched (Ptc). Combined depletion of both serotonin and norepinephrine with para-chlorophenylalanine (PCPA) resulted in a significant decrease in Smo and Ptc mRNA within the dentate gyrus subfield of the hippocampus. However, selective depletion of serotonin, using the serotonergic neurotoxin 5,7-dihyrdroxytryptamine (5,7-DHT), or norepinephrine, using the noradrenergic neurotoxin DSP-4, did not alter expression of Shh and its co-receptors, Smo and Ptc. Acute treatment with the monoamine releasing agent, para-chloroamphetamine (PCA) significantly upregulated Smo mRNA within the dentate gyrus. However, acute or chronic treatment with pharmacological antidepressants that modulate monoaminergic neurotransmission did not regulate Shh cascade expression. These results indicate that robust changes in monoamine levels can regulate the expression of the Shh signaling cascade in the adult rodent brain.

Animal models of depression exhibit decreased adult hippocampal neurogenesis [19,27], whereas sustained antidepressant administration increases hippocampal neurogenesis [18] and is thought to contribute to the behavioral effects of antidepressants [23]. The monoaminergic hypothesis suggests that decreased monoamines underlie the pathogenesis of depression, and increased monoamines contribute to antidepressant action [6]. Recently monoaminergic neurotransmission has been implicated in the effects of depression models and antidepressants on adult hippocampal neurogenesis [23,26]. A decline in hippocampal neurogenesis is observed following serotonin (5-HT) and/or norepinephrine (NE) depletion, while increased hippocampal neurogenesis is seen following elevation of monoamine levels [4,9,12,18]. However, the pathways that underlie the effects of monoamines on adult hippocampal neurogenesis are not understood.

Sonic hedgehog (Shh) is a powerful regulator of adult hippocampal neurogenesis and is essential for the maintenance of the adult stem cell niches [14,17,21]. In the adult brain, Shh is expressed in the neocortex and the horizontal and vertical limbs of the diagonal band from where it is thought to be anterogradely transported to the hippocampus [24,25]. Within the hippocampus, expression of the Shh co-receptors Patched (Ptc) and Smoothened (Smo) is seen in the dentate gyrus subfield including the neurogenic niche of subgranular zone (SGZ) [14,24]. Smo protein has also been recently shown to localize predominantly to mossy fiber nerve terminals in the hippocampus [20]. Recent evidence indicates that the neurogenic effects of electroconvulsive seizure (ECS) treatment, one of the most robust forms of antidepressant therapy, requires Shh signaling and ECS also regulates the hippocampal expression of Smo and Ptc [1]. We hypothesized that monoaminergic neurotransmission may influence the expression of the Shh cascade. The aim of the present study was to address whether robust perturbations in the levels of 5-HT and NE or treatment with pharmacological antidepressants regulate the expression of key components of the Shh signaling cascade.

Male Sprague–Dawley rats (200–250 g; TIFR animal breeding colony) were group-housed and maintained on a controlled 12 h light/dark cycle with access to food and water *ad libitum*. All animal procedures were carried out in accordance with the NIH *Guide for the Care and Use of Laboratory Animals*, and were approved by the TIFR Institutional Animal Ethics Committee.

To induce a combined depletion of norepinephrine (NE) and serotonin (5-HT), animals were treated with p-chlorophenylalanine (PCPA, 300 mg/kg—2 days, 100 mg/kg—2 days; Sigma, USA) as previously described [4,9]. To induce a selective depletion of NE, animals were treated with N-Ethyl-N-(2-chloroethyl)-2-bromobenzylamine hydrochloride (DSP-4, 10 mg/kg, Sigma) using a previously described protocol [12]. To induce a selective depletion of 5-HT, animals received an intracerebroventricular (i.c.v.) infusion (AP-0.8 mm, ML-1.4 mm and DV-4.0 mm from Bregma [22]) of 5,7-dihydroxytryptamine (5,7-DHT, 200 μg/animal, 20 μg/μl, Sigma) as reported previously [9]. The choice of dose, treatment paradigm and time-point for sacrifice for the above studies was based on prior literature that indicated a robust depletion of the specific monoamines [4,9,12]. To induce a robust monoamine release, animals received p-Chloroamphetamine (PCA, 10 mg/kg, Sigma) or vehicle (0.9% saline) through intraperitoneal (i.p.) injection and were sacrificed 2 h later. For the acute antidepressant experiment, animals received an i.p. injection of fluoxetine (FLX; 5 mg/kg), tranylcypromine (TCP; 7.5 mg/kg), desipramine (DMI; 15 mg/kg) or vehicle (0.9% saline) and were sacrificed 2 h later. For the chronic antidepressant experiment, animals received i.p. injections of FLX (5 mg/kg), TCP (7.5 mg/kg for 7 days and 10 mg/kg for 14 days), DMI (15 mg/kg) or vehicle (0.9 % saline) once daily for 21 days and were sacrificed 2 h after the last treatment. The choice of doses for antidepressant treatments was based on prior literature that indicated an effect on adult hippocampal progenitor proliferation at these doses [18]. All antidepressant drugs were obtained from Sigma. After decapitation, brains were frozen prior to cryostat sectioning and *in situ* hybridization analysis.

*In situ* hybridization was carried out as previously described [1] on 14 μm thick coronal sections. Slides were fixed, acetylated and dehydrated. Rat Shh, Smo and Ptc cRNA probes were transcribed using ^35^S-labeled UTP (Amersham) from plasmids provided by Dr. Martial Ruat (CNRS, France). Slides were incubated with radiolabeled riboprobes (1 × 10^6^ cpm/150 μl per slide) in hybridization buffer overnight and then subjected to stringent post-hybridization washes. Slides were exposed to Hyperfilm β-max (Amersham) for 2–3 weeks. Shh, Ptc or Smo sense riboprobes did not yield significant hybridization (data not shown) confirming the specificity of the signal observed with the antisense riboprobes. Shh, Ptc and Smo mRNA levels were quantitated with the Macintosh-based Scion Image software (Scion, USA) using ^14^C standards (Amersham, USA) for calibration and to correct for non-linearity. For Shh mRNA, expression in the horizontal and vertical limbs of the diagonal band (VDB) and in the neocortex was quantitated. For Ptc and Smo mRNA, the expression in the dentate gyrus was determined. The optical density was determined by outlining an equivalent area in each section, and the means were determined from measurements taken on both sides of 4 individual sections/animal.

Quantitative real-time PCR (qPCR) was also performed for acute PCA treatment. Animals (*n* = 5/group) were treated with PCA or vehicle and sacrificed as described earlier. Hippocampi were subdissected in cold saline and frozen in liquid nitrogen. Total RNA was isolated from hippocampal tissue using Tri Reagent (Sigma), according to the manufacturer's protocol. RNA was quantified using Nanodrop (Eppendorf, Germany) and RNA quality determined by resolving on 1% formaldehyde agarose gel. cDNA was synthesized from 200 ng of total RNA per sample using Quantitect reverse transcription kit (Qiagen, USA), as per the manufacturer's protocol. cDNA was amplified and visualized using a SYBR Green kit (Applied Biosystems, USA) in a Realplex mastercycler (Eppendorf). Hypoxanthine phopshoribosyl transferase (HPRT) was used as the endogenous control. To compare HPRT and target gene, relative quantification was performed using comparative *C*_T_ method [2]. Briefly, this comparative *C*_T_ method involved averaging duplicate samples of each target and endogenous control in both control and treatment samples [i.e. Δ*C*_T_ = absolute *C*_T_ value − endogenous control *C*_T_ value and ΔΔ*C*_T_ = Δ*C*_T_ treatment − Δ*C*_T_ control]. The fold change was calculated according to the formula $2^{\left(-\Delta \Delta C_{\text{T}}\right)}$. The primer sequences used were Ptc F—CCATTTCTTGCCCTTGGTGTTGGT, R—AATGCAGCCATGAAGAAGGCAGTG; Smo F—AATTGGCCTGGTGCTTATTGTGGG, R—AGGGTGGTTGCTCTTGATGGAGAA; HPRT F—GCAGACTTTGCTTTCCTTGG, R—GTCTGGCCTGTATCCAACACT.

Experiments were analyzed for differences using the unpaired Student's *t*-test (Prism, Graphpad, USA) with significance determined at *p* values < 0.05.

Combined, but not selective, depletion of norepinephrine and serotonin levels downregulates Smo and Ptc mRNA levels in the dentate gyrus.

The expression of Smo, the signaling component of the Shh receptor complex, was found to be significantly decreased (∼30%) in the hippocampal dentate gyrus subfield following depletion of both NE and 5-HT by PCPA treatment. In addition, there was a small but significant decrease (∼20%) in the level of Ptc mRNA in the DG of PCPA treated animals as compared to the vehicle group (Fig. 1). In striking contrast, selective noradrenergic (DSP-4) and serotonergic (5,7-DHT) neurotoxin treatments did not alter Smo or Ptc mRNA expression in the hippocampus (Fig. 2). The mRNA expression of the ligand Shh in the VDB (Figs. 1 and 2) and the neocortex (PCPA treatment: Veh = 100 ± 3.27, PCPA = 98.83 ± 2.09; DSP-4 treatment: Veh = 100 ± 11.47, DSP-4 = 99.75 ± 7.22; 5,7-DHT treatment: Veh = 100 ± 5.82, 5,7-DHT = 113.09 ± 4.29; results are expressed as percent of Vehicle and are the mean ± S.E.M.) was not influenced by PCPA, DSP-4 or 5,7-DHT treatments. As previously reported [24], Shh mRNA expression was high in the VDB and was expressed at relatively lower levels in the neocortex (layer V).

Treatment with the monoamine releasing agent, PCA, increases Smo mRNA in the dentate gyrus, however pharmacological antidepressants do not regulate the Shh signaling cascade.

Acute PCA treatment resulted in a robust and significant upregulation of Smo mRNA (∼65%) within the DG subfield of the hippocampus (Fig. 1). PCA treatment did not influence either the expression of Shh in the basal forebrain (Fig. 1) or neocortex (PCA Treatment, Shh mRNA levels: Veh = 100 ± 8.26, PCA = 113.35 ± 11.25, results are expressed as percent of Vehicle and are the mean ± S.E.M.) or the co-receptor Ptc in the hippocampal subfields (Fig. 1). The *in situ* regulation of Smo and Ptc mRNA were further validated by qPCR for acute PCA treatment. Acute PCA resulted in a 70% increase in Smo mRNA in the hippocampus as compared to vehicle treated controls (Δ*C*_T_ acute PCA = 0.915 ± 0.1762; Δ*C*_T_ Vehicle = 1.68 ± 0.2463; ΔΔ*C*_T_ = −0.765; results are expressed as Δ*C*_T_ and are the mean ± S.E.M.; *p* < 0.05, Student's *t*-test). Hippocampal Ptc mRNA levels were found to be unaltered following acute PCA treatment (Δ*C*_T_ acute PCA = 3.041 ± 0.3414; Δ*C*_T_ Vehicle = 3.577 ± 0.2124; ΔΔ*C*_T_ = −0.536; results are expressed as Δ*C*_T_ and are the mean ± S.E.M.; *p* > 0.05, Student's *t*-test). Further, Shh mRNA levels in the VDB and the neocortex, and Smo and Ptc mRNA levels in the DG were not altered by either acute or chronic treatment with the pharmacological antidepressants DMI, FLX or TCP (Table 1).

The present study indicates that robust changes in levels of both 5-HT and NE modulate the expression of the Shh co-receptors, Smo and Ptc, in the hippocampal dentate gyrus subfield. While depletion of both 5-HT and NE (PCPA) decreased Smo and Ptc hippocampal mRNA levels, selective depletion of either 5-HT (5,7-DHT) or NE (DSP-4) did not alter their expression. Further, acute treatment with the monoamine releasing agent PCA significantly increased Smo mRNA levels. In contrast, acute or chronic antidepressant treatment with the serotonin selective reuptake inhibitor, fluoxetine, the norepinephrine selective reuptake inhibitor, desipramine, and the monoamine oxidase inhibitor, tranylcypromine did not regulate the expression of the Shh cascade. Our study provides novel evidence of an influence of NE and 5-HT on the expression of the Shh signaling cascade, a major developmental pathway that continues to be expressed in the adult brain.

The effects of Shh have been best studied during development, where it regulates the proliferation, survival and fate determination of embryonic neuronal and glial precursors [7]. Recently Shh signaling has been shown to play a critical role in maintaining stem cell niches in the adult brain [17], where it regulates adult progenitor proliferation [14,21]. These studies suggest that the Shh pathway may continue to have an important role in adulthood in regulating structural plasticity. Given prior evidence that monoamines regulate the proliferation and survival of hippocampal progenitors [4,9,10,12,16], our results raise the possibility that the effects of monoaminergic neurotransmission on the expression of the Shh signaling cascade could contribute to the influence of monoamines on adult neurogenesis.

In this regard, it is particularly interesting to note that selective depletion of NE (DSP-4) or 5-HT (5,7-DHT) does not alter expression of the Shh cascade whereas combined depletion of both monoamines (PCPA) results in a significant decline in hippocampal Smo and Ptc mRNA levels. While DSP-4 and 5,7-DHT selectively induce a decrease in the proliferation of hippocampal progenitors [4,12], PCPA has a more dramatic effect on hippocampal neurogenesis resulting in robust decreases in both proliferation and survival of adult hippocampal progenitors [9]. It is possible to speculate a role for reduced Shh signaling in mediating the robust decline in neurogenesis following PCPA administration. Strikingly, treatment with PCA, which would increase the levels of both NE and 5-HT [11], resulted in a significant increase in the expression of Smo mRNA. We did not observe any regulation of Shh mRNA expression by either PCPA or PCA suggesting the possibility that monoamine neurotransmission may modulate expression of receptors for Shh directly rather than via an effect on the ligand. Future studies are required for a clear understanding of the role of the Shh cascade in mediating the effects of monoaminergic perturbations on adult hippocampal neurogenesis.

It is intriguing that ECS and PCA differ in their effects on hippocampal Smo mRNA expression, as ECS would also be expected to enhance monoamine levels within the hippocampus [8]. Given that ECS induces the release of several other neurotransmitters besides monoamines [5,15], it points to the possibility that other neurotransmitters pathways and neuronal activity may induce a different effect on Smo mRNA expression than that observed with selective monoamine release induced by acute PCA treatment.

At present the mechanism by which NE and 5-HT may influence expression of the Shh signaling cascade is unclear. However, the cAMP-Protein kinase A (PKA) pathway, is an attractive candidate. Activation of the cAMP cascade by the β adrenoceptor agonist isoproterenol enhances Ptc expression in the pineal gland [3]. It is possible to speculate that the decreased Smo and Ptc expression following robust monoamine depletion may involve decreased cAMP cascade activation. Taken together, these studies raise the possibility that monoaminergic signaling can modulate the gene expression of the Shh pathway in adulthood. Given that Shh plays a critical role during development, further studies to explore the effects of monoaminergic perturbation during gestation on fetal Shh signaling and consequences on brain development are warranted.

Unlike previous results [1] that indicate a significant effect of ECS treatment on hippocampal Smo and Ptc expression and a role for Shh signaling in the neurogenic effects of ECS, the present study indicates that acute or chronic pharmacological antidepressant treatments do not regulate Shh cascade expression. Interestingly, PCA, which robustly enhances monoamine levels, did upregulate Smo mRNA expression, however pharmacological antidepressants, which also elevate synaptic monoamine levels did not appear to do so. This discrepancy in the regulation of Smo mRNA may be a consequence of the extent of elevation in monoamine levels observed with a monoamine releasing agent [13,28] in contrast to the change seen with agents that block reuptake or degradation. This points to the possibility that modulation of the hippocampal expression of the Shh cascade may require very robust changes in both 5-HT and NE. This is supported by evidence that ECS, PCA and PCPA treatments can regulate Ptc and Smo expression, whereas selective depletions of 5-HT or NE and treatment with pharmacological antidepressants does not regulate the Shh cascade expression.

In conclusion, our results provide novel evidence that monoaminergic neurotransmission modulates the Shh signaling cascade expression in the adult mammalian brain, and highlight the need for future studies to understand the mechanism of this regulation and the role of Shh signaling in the effects of monoamines on adult structural plasticity.

## Figures and Tables

**Fig. 1:**
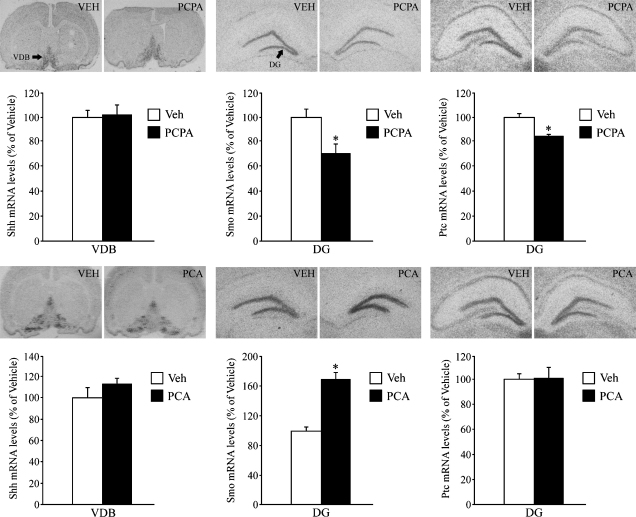
Influence of PCPA & PCA treatment on the expression of the Shh cascade in the adult rat brain. Rats were administered either PCPA, PCA or vehicle (VEH) treatment in separate experiments, and levels of Shh, Smo and Ptc mRNA were determined by *in situ* hybridization and quantitative densitometry. PCPA treatment decreased the expression of Smo and Ptc mRNA in the dentate gyrus (DG), but did not influence Shh mRNA expression in the Horizontal limb/Vertical limb of the Diagonal Band (VDB)—Upper Panel. PCA treatment significantly increased the levels of Smo mRNA in the DG, but did not alter the levels of Ptc mRNA in the DG or Shh in the VDB/HDB region—Lower Panel. Representative autoradiographs of Shh, Smo and Ptc mRNA from vehicle and PCPA treated animals (Upper Panel) and vehicle and PCA treated animals (Lower Panel) are shown. Results are expressed as percent of vehicle and are the mean ±S.E.M. (*n* = 5 /group); ^*^p < 0.05 as compared with vehicle (Student's *t*-test).

**Fig. 2:**
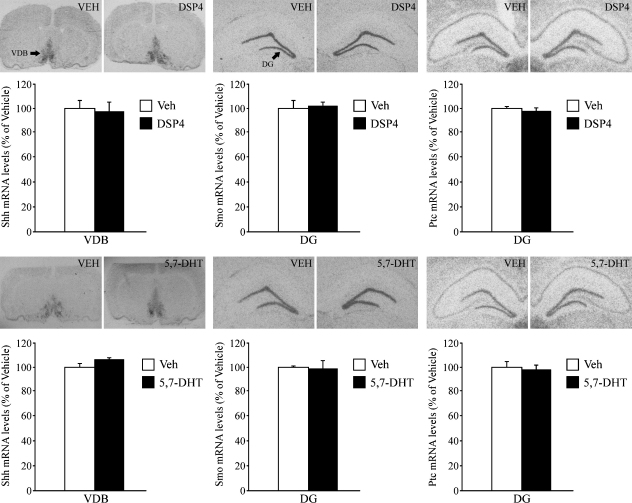
Influence of DSP-4 and 5,7-DHT treatment on the expression of the Shh cascade in the rat brain. Rats received treatment with vehicle (VEH), DSP-4 or 5,7-DHT in separate experiments, and levels of Shh, Smo and Ptc mRNA were determined with *in situ* hybridization and quantitative densitometry. DSP-4 or 5,7-DHT treatment did not influence Shh mRNA expression in the Horizontal limb/Vertical limb of the Diagonal Band (VDB) or the expression of the Shh receptors, Smo and Ptc in the hippocampal dentate gyrus (DG) subfield. Representative autoradiographs of Shh, Smo and Ptc mRNA from vehicle and DSP-4 (Upper Panel), and vehicle and 5,7-DHT (Lower Panel) treated animals are shown. Results are expressed as percent of vehicle and are the mean ±S.E.M. (*n* = 5/group).

**Table 1:** Acute or chronic treatment with different classes of antidepressants does not influence the mRNA expression of the Shh cascade.

		Shh mRNA (Neocortex)	Shh mRNA (VDB/HDB)	Ptc mRNA (DG)	Smo mRNA (DG)
Acute antidepressant treatment	VEH	100 ± 13.45	100 ± 15.44	100 ± 4.94	100 ± 11.42
	Ac DMI	108.03 ± 1.31	98.66 ± 7.04	110.9 ± 7.43	93.88 ± 12.89
	VEH	100 ± 4.08	100 ± 13.34	100 ± 5.81	100 ± 4.67
	Ac FLX	100.88 ± 1.7	96.66 ± 3.09	92.92 ± 2.17	90.74 ± 25.84
	VEH	100 ± 3.1	100 ± 13.34	100 ± 4.94	100 ± 12.6
	Ac TCP	91.1 ± 0.87	89.06 ± 5.92	93.16 ± 5.14	86.58 ± 2.17

Chronic antidepressant treatment	VEH	100 ± 10.27	100 ± 12.61	100 ± 8.5	100 ± 10.1
	Chr DMI	72.15 ± 8.55	81.68 ± 9.67	112.46 ± 5.82	91.56 ± 10.6
	VEH	100 ± 6.18	100 ± 13.55	100 ± 7.36	100 ± 3.94
	Chr FLX	91.36 ± 8.82	80.32 ± 2.5	97.44 ± 4.59	115.35 ± 16.4
	VEH	100 ± 6.18	100 ± 13.55	100 ± 7.38	100 ± 11.25
	Chr TCP	92.3 ± 9.16	77.99 ± 9.78	113.40 ± 0.58	78.38 ± 10.66

Animals were administered Desipramine (DMI), Fluoxetine (FLX) or Tranylcypromine (TCP) either as a single dose (acute) or once daily for 21 days (chronic), and the mRNA levels of Shh, Smo and Ptc were studied using *in situ* hybridization and analyzed by quantitative densitometry. Shh mRNA levels in the HDB/VDB region and in the neocortex were unaltered after either acute or chronic treatment by DMI, FLX or TCP. Smo and Ptc mRNA levels in the hippocampal DG were also unaffected by acute or chronic treatment with these agents. Results are expressed as percent of vehicle and are the mean ±S.E.M. (*n* = 4–5/group).
